# Exposure to Perchlorate in Lactating Women and Its Associations With Newborn Thyroid Stimulating Hormone

**DOI:** 10.3389/fendo.2018.00348

**Published:** 2018-07-03

**Authors:** Yasemin Ucal, Ozlem N. Sahin, Muhittin Serdar, Ben Blount, Pinar Kumru, Murat Muhcu, Mustafa Eroglu, Cansu Akin-Levi, Z. Zeynep Yildirim Keles, Cem Turam, Liza Valentin-Blasini, Maria Morel-Espinosa, Mustafa Serteser, Ibrahim Unsal, Aysel Ozpinar

**Affiliations:** ^1^Department of Medical Biochemistry, School of Medicine, Acibadem Mehmet Ali Aydinlar University, Istanbul, Turkey; ^2^Department of Pediatrics, School of Medicine, Acibadem Mehmet Ali Aydinlar University, Istanbul, Turkey; ^3^Centers for Disease Control and Prevention, Atlanta, GA, United States; ^4^Department of Obstetrics and Gynecology, Zeynep Kamil Research and Training Hospital, Istanbul, Turkey; ^5^Department of Obstetrics and Gynecology, Haydarpasa Hospital of Gülhane Military Practice School and Hospital, Istanbul, Turkey; ^6^Department of Obstetrics and Gynecology, Haydarpasa Numune Training and Research Hospital, Istanbul, Turkey; ^7^Acibadem LabMed Laboratories, Istanbul, Turkey

**Keywords:** NIS inhibitors, perchlorate, nitrate, thiocyanate, colostrum, newborn thyroid health

## Abstract

**Background:** Perchlorate, thiocyanate, and nitrate can block iodide transport at the sodium iodide symporter (NIS) and this can subsequently lead to decreased thyroid hormone production and hypothyroidism. NIS inhibitor exposure has been shown to reduce iodide uptake and thyroid hormone levels; therefore we hypothesized that maternal NIS inhibitor exposure will influence both maternal and newborn thyroid function.

**Methods:** Spot urine samples were collected from 185 lactating mothers and evaluated for perchlorate, thiocyanate, and nitrate concentrations. Blood and colostrum samples were collected from the same participants in the first 48 h after delivery. Thyroid hormones and thyroid-related antibodies (TSH, fT3, fT4, anti-TPO, anti-Tg) were analyzed in maternal blood and perchlorate was analyzed in colostrum. Also, spot blood samples were collected from newborns (*n* = 185) between 48 and 72 postpartum hours for TSH measurement. Correlation analysis was performed to assess the effect of NIS inhibitors on thyroid hormone levels of lactating mothers and their newborns in their first 48 postpartum hours.

**Results:** The medians of maternal urinary perchlorate (4.00 μg/g creatinine), maternal urinary thiocyanate (403 μg/g creatinine), and maternal urinary nitrate (49,117 μg/g creatinine) were determined. Higher concentrations of all three urinary NIS inhibitors (μg/g creatinine) at their 75th percentile levels were significantly correlated with newborn TSH (*r* = 0.21, *p* < 0.001). Median colostrum perchlorate level concentration of all 185 participants was 2.30 μg/L. Colostrum perchlorate was not significantly correlated with newborn TSH (*p* > 0.05); however, there was a significant correlation between colostrum perchlorate level and maternal TSH (*r* = 0.21, *p* < 0.01). Similarly, there was a significant positive association between colostrum perchlorate and maternal urinary creatinine adjusted perchlorate (*r* = 0.32, *p* < 0.001).

**Conclusion:** NIS inhibitors are ubiquitous in lactating women in Turkey and are associated with increased TSH levels in newborns, thus signifying for the first time that co-exposure to maternal NIS inhibitors can have a negative effect on the newborn thyroid function.

## Introduction

The thyroid hormones T4 and T3 are essential for normal development and survival of the human newborn. In infants, chronically low levels of thyroid hormones can lead to mental deficiency and motor deficits such as spasticity, dystonia or rigidity (1). TSH, which is secreted from the anterior pituitary gland, stimulates iodine trapping, thyroid hormone production, and thyroid hormone release from the thyroid gland. Thyroid hormone production requires adequate iodine intake, as iodine is a critical component of T4 and T3. The sodium iodide symporter (NIS) is unique in that it transports iodine into the thyroid gland of mother and fetus, as well as the newborn child. Additionally, NIS is also expressed at the lactating breast and mediates active transport of iodine into the milk for thyroid hormone synthesis by the newborn (2). Several environmentally-derived competitive NIS inhibitors, particularly perchlorate, thiocyanate, and nitrate, are known to inhibit both T4 and T3 production and lead to increased TSH production (3, 4). In the presence of environmentally-derived inhibitors, they can also be actively transported into the breast milk and subsequently be consumed by infants. Therefore, exposure of lactating women to perchlorate, thiocyanate, and nitrate may affect both maternal and newborn thyroid function and health.

Perchlorate is an inorganic ion that is widely manufactured for use in rocket propellant systems, matches, fireworks, and other industrial applications (5, 6). Perchlorate can also form naturally in the atmosphere and accumulate in the soil of arid regions (5). Moreover, perchlorate from both natural and manufactured sources can be concentrated in foods such as green vegetables, water, eggs, and milk (7). Human perchlorate exposure arises when perchlorate-contaminated food and drinking water are consumed (8). Thiocyanate is the primary metabolite of hydrogen cyanide in cigarette smoke and also can be formed after digestion of some plant-based foods (9). Nitrates derived from agricultural fertilizer runoff or exposure to sewage are commonly found in food and drinking water (10). Thiocyanate and nitrate less effectively inhibit iodide transport than perchlorate (11).

Thyroid health is an important global issue since mild iodine deficiency is prevalent in many parts of the world, including some European countries, the USA and Australia (12) and exposure to NIS inhibitors is ubiquitous (13–16). Additionally, Turkey has mild iodine deficiency (17) and a high intake of the iodide uptake inhibitor perchlorate is detected in Turkish populations (15). Because information on the effect of the NIS inhibitors on lactating women and newborns is limited in the literature, we proposed to study this in Turkish women and their newborns (18–20). To our knowledge, this is the first study to evaluate exposure to NIS inhibitors in lactating women and the association between perchlorate exposure and newborn TSH levels.

## Materials and methods

### Recruitment of subjects

Between June 2013 and August 2013, 258 healthy pregnant women with a singleton pregnancy were recruited at routine examinations in week 38 at the state hospital specialized in women obstetrics and gynecology—Zeynep Kamil Research and Training Hospital—in Istanbul, Turkey. This state hospital was purposefully selected because it is one of the major obstetrics and gynecology hospitals in Turkey and provides a large sample-size of healthy pregnant women. An experienced doctor recruited healthy pregnant women in the clinic. Any woman with a past diagnosis of thyroid disorders, chronic illnesses, use of thyroid-active medication (e.g., amiodarone, glucocorticoids, dopamine, propranolol, iodine, lithium, phenytoin, carbamazepine) was excluded. Of the 258 eligible pregnant women, 200 met inclusion criteria and gave informed consent. Informed consent was obtained at the time of enrollment of the women in the study. Only women who could provide a sufficient amount of colostrum (~5 ml), were included in the study. Since sufficient colostrum to measure perchlorate was obtained for only 185 samples, 15 women were eliminated from the original sample.

All subjects resided in suburban Istanbul and provided standardized information on their medical histories and lifestyles, including cigarette smoke exposure, diabetes history, age, residency, and family medical history. This study was approved by Acibadem University Ethics Committee (ATADEK 2013-507).

### Sample collection

**Figure 1:**
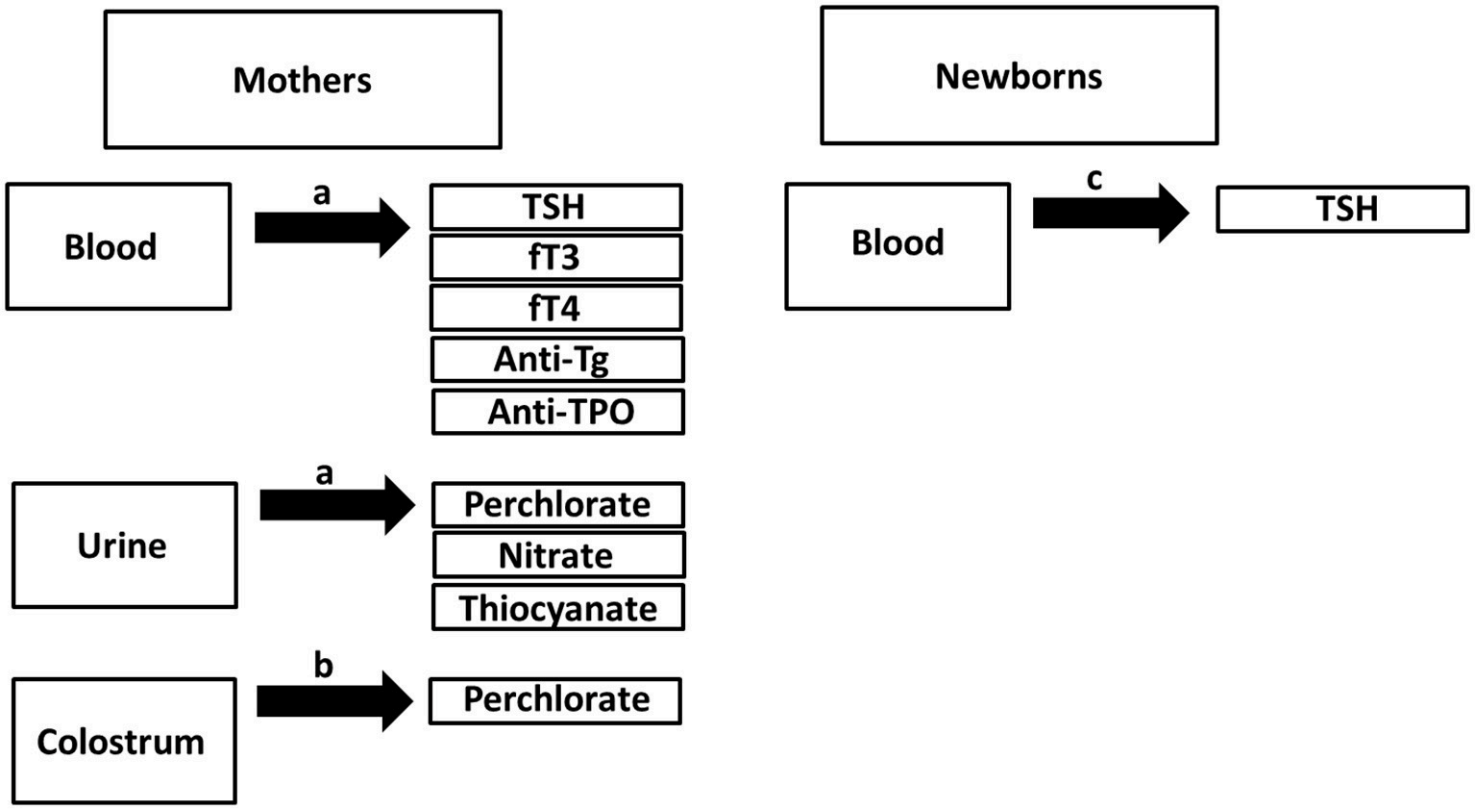
Overview of sample collection. ^a^Sample collection in the first 48 post-partum hours. ^b^Sample collection either in the first 72 postpartum hours for subjects having C-section or 48 postpartum hours for subjects having vaginal delivery. ^c^Spot blood collection using heel prick test >48th postpartum hours.

Samples were obtained between June and August 2013. In the first 48 postpartum hours, urine samples were collected from mothers using standard urine collection containers, as described previously (15), while blood samples were obtained in blood serum tubes. Colostrum samples were collected from the 185 lactating women as soon as lactation began, with the timing being within 72 postpartum hours for subjects having C-section and within 48 postpartum hours for subjects having spontaneous vaginal delivery. Colostrum samples were collected into sterile sample collection cups via manual expression. Additionally, newborn heel-prick blood was obtained between 48 and 72 h after delivery to measure newborn TSH levels. Colostrum, urine, serum, and newborn serum samples were kept at −80°C until analysis. Sample collection procedures are detailed in Figure 1.

### Laboratory measurements and calculations

Perchlorate, thiocyanate, and nitrate levels were measured in maternal urine samples while perchlorate was additionally measured in colostrum samples. Thiocyanate is unstable in colostrum, and therefore not measured (B. Blount, personal communication). Analyses of maternal urinary perchlorate, thiocyanate, nitrate, and colostrum perchlorate were performed on triple quadrupole ion chromatography-mass spectrometry at the Centers for Disease Control and Prevention. The method was modified from Valentin-Blasini et al. (21). A modified version of the Jaffe method was used for measuring urinary creatinine concentrations (Roche Diagnostics Corp., IN, USA) (22). Creatinine excretion rates were calculated using the method detailed in our previous publication (15). All reported data complied with rigorous quality control procedures of the CDC Division of Laboratory Sciences (23).

Maternal fT3, fT4, TSH, anti-thyroid peroxidase (TPO) antibodies, and anti-thyroglobulin (anti-Tg) antibodies from serum samples were determined by electro-chemiluminescence immunoassay (ECLIA) using Elecsys 2010 (Roche Diagnostics, Germany).

Newborn perchlorate intake was estimated based on the perchlorate concentrations in breast milk from the US Environmental Protection Agency (EPA) Exposure Handbook and reported rates of breast milk consumption by newborns as 100–120 ml/kg/day (24).

### Statistical analysis

We used descriptive statistics (mean, median, first and third quartiles, and standard deviation) to characterize subjects' demographic and clinical variables. Continuous values were represented as medians and interquartile ranges (IQR). Shapiro-Wilk test was applied to determine data normality. Subjects' data were examined by Pearson and Spearman correlations using Analyse-it Software (Analyse-it Software, Ltd. United Kingdom, V4.20.1). The relationship between subjects' demographic and clinical variables or between the two variables and NIS inhibitors were performed using correlation analysis. Bonferroni correction was applied to reduce type I errors in the multiple tests.

Maternal co-exposure to NIS inhibitors was assessed by establishing subgroups in the study population. For this purpose, subjects with higher than 75% maternal urinary NIS inhibitor concentration were selected and assigned to four groups: those having three NIS inhibitors elevated, those having two, those having just one, and those have none Statistical significance between four groups was determined by one-way analysis of variance (ANOVA) followed by Bonferroni's *post-hoc* test, considering *p* ≤ 0.05 as significantly different.

## Results

### Demographic characteristics and thyroid function test results

The primary study participant characteristics [i.e., maternal age, body mass index (BMI), maternal TSH, maternal fT3, maternal fT4, maternal anti-TPO and anti-Tg levels] are summarized in Table 1. Newborn TSH levels, birth weights and estimated perchlorate intake levels are given in Table 2. FT4 was negatively correlated with the BMI of the 185 participants (*r* = −0.20, *p* = 0.01). In addition, there was a negative trend between newborn TSH and maternal fT3.

**Table 1:** Maternal characteristics and maternal thyroid hormone function tests (*n* = 185).

**Variable**	**Mean**	***SD***	**25th percentile**	**Median**	**75th percentile**
Age	27.08	5.63	23.00	27.00	30.00
BMI^ϕ^ (kg/m^2^)	29.10	4.50	25.80	28.70	32.00
TSH (μU/ml)	2.56	1.94	1.40	2.20	3.10
fT3 (pmol/L)	4.52	0.75	4.00	4.30	4.80
fT4 (pmol/L)	12.06	2.04	10.70	12.00	13.20
AntiTPO (IU/ml)	13.98	35.88	5.00	6.00	8.40
Anti TG (IU/ml)	30.19	85.50	12.20	16.90	21.40

ϕ *18.50 < BMI < 24.90 defined as normal weight, 25.00 < BMI < 29.90 defined as overweight, and BMI ≥30.0 defined as obese (25). For TSH(μU/ml) measurement, the reference intervals were 0.25–4,550 μU/ml, inter assay variation of the test was 3.62–4.28 and total coefficient of variation CV (%) was 5.13–6.64. For fT3 (pmol/L) measurement, the reference intervals were 3.50–6.50, inter assay variation of the test was 2.47–3.08, total CV (%) was 2.76–4.05. For fT4 (pmol/L) measurement, the reference intervals were 11.50–22.70, inter assay variation of the test was 2.33–4.00, total CV (%) was 3.44–4.58*.

**Table 2:** Newborn characteristics, TSH concentrations and perchlorate intake estimations (*n* = 185).

**Variable**	**Mean**	***SD***	**25th Percentile**	**Median**	**75th Percentile**
Birth weight (g)	3,204	502	2,960	3,160	3,520
TSH (μU/ml)^ϕ^	4.62	3.26	2.50	3.80	6.10
Perchlorate intake estimation^¥^ (μg/kg/day)	0.20	0.23	0.00	0.10	0.30

ϕ Newborn TSH concentration was measured in dry blood spots by heel prick test.

¥ *Author's calculations using U.S EPA Exposure Handbook estimation about newborn breast milk consumption rate of 100–120 ml/kg/day (24)*.

### Association between maternal urinary NIS inhibitors and maternal thyroid function test results

The median maternal urinary perchlorate (4.00 μg/g creatinine), maternal urinary thiocyanate (403 μg/g creatinine) and maternal urinary nitrate (49,117 μg/g creatinine) were determined (Table 3). Urinary nitrate was strongly correlated with both thiocyanate (*r* = 0.36, *p* < 0.0001) and perchlorate (*r* = 0.44; *p* < 0.0001).

**Table 3:** Maternal urinary NIS inhibitor concentrations and maternal colostrum perchlorate concentration (*n* = 185).

**Variable**	**Mean**	***SD***	**25th percentile**	**Median**	**75th percentile**
Urinary perchlorate (μg/g creatinine)	5.60	5.69	2.30	4.00	6.30
Urinary thiocyanate (μg/g creatinine)	610	802	207	403	686
Urinary nitrate (μg/g creatinine)	62,289	62,901	34,228	49,117	67,881
Colostrum perchlorate (μg/L)	4.09	4.75	1.20	2.30	5.70

For the 185 women, there was no correlation between maternal TSH and any creatinine-adjusted NIS inhibitor levels. Additionally, there was no statistically significant difference between co-exposure to maternal NIS inhibitors and maternal TSH.

There were no significant correlations between free thyroid hormones (fT3 and fT4) and any maternal urinary NIS inhibitors nor between thyroid auto-antibodies (anti-TPO and anti-Tg) and any NIS inhibitors.

### Association between maternal urinary NIS inhibitors and newborn TSH

**Figure 2:**
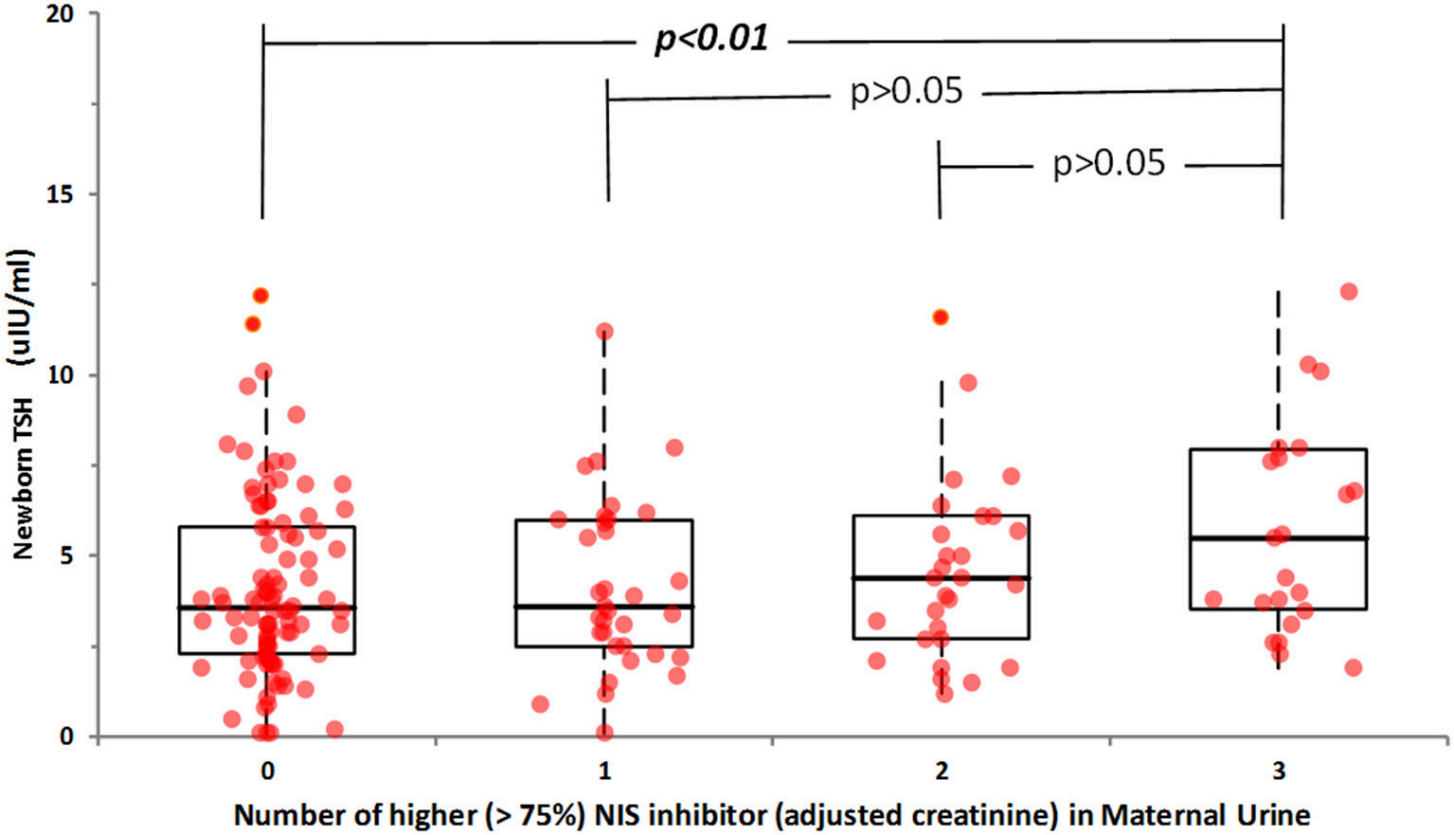
Relationship between newborn TSH and co-exposure to maternal urinary NIS inhibitors. Subjects with higher than 75% maternal urinary NIS inhibitor concentration were selected and assigned to four groups: those having three NIS inhibitors elevated (Group 3), those having two (Group 2), those having just one (Group 1), and those have none (Group 0). Statistically significant difference was obtained only when co-exposure to three NIS inhibitors at their highest percentile occurred.

For the 185 newborns, a significant correlation was seen between newborn-screen TSH and maternal urinary perchlorate (*r* = 0.24, *p* < 0.001) but not with maternal urinary thiocyanate and nitrate concentrations. However, there was no significant correlation between newborn TSH and any creatinine-adjusted maternal urinary NIS inhibitors. Nevertheless, when newborn TSH and 75th percentile creatinine-adjusted urinary perchlorate, thiocyanate and nitrate levels of lactating women were compared in a regression analysis model, women with the highest quartile urinary concentrations of all 3 NIS inhibitors had newborns with higher newborn TSH levels (*r* = 0.21, *p* < 0.001) (Figure 2).

### Maternal perchlorate concentration in colostrum and its association with maternal and newborn TSH

**Figure 3:**
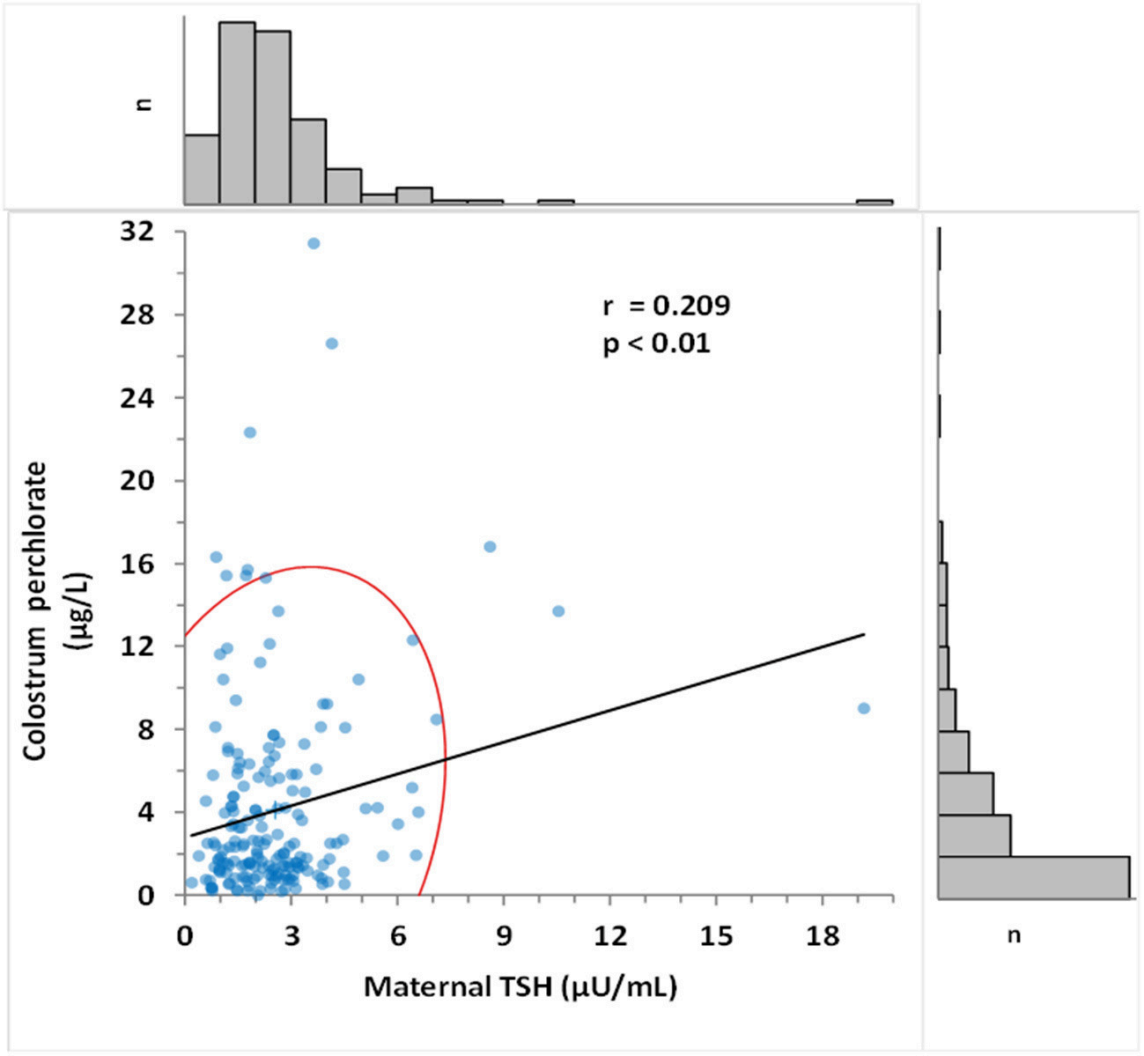
Correlation between colostrum perchlorate levels and maternal TSH. Pearson's correlation coefficient was determined as 0.209 (*p* < 0.015). Frequency histogram shows the number of values (n) in the corresponding axis. The red curve represents 95% CI of the distribution.

The median maternal perchlorate concentration in colostrum was 2.30 μg/L. Calculation of estimated newborn perchlorate intake (24) revealed a median dose of 0.10 μg/kg/day. Colostrum perchlorate concentration was significantly correlated with maternal urinary creatinine-adjusted perchlorate (*r* = 0.32, *p* < 0.001) and with maternal TSH (*r* = 0.21, *p* < 0.01) (Figure 3), but not with newborn TSH.

## Discussion

The present study represents the first assessment of NIS inhibitor exposure in lactating women and their newborns in Turkish populations. Results from this study showed that NIS inhibitors were ubiquitous in lactating women. Additionally, lactating women with the highest quartile urinary concentrations of all 3 NIS inhibitors had newborns with higher newborn TSH levels. Previous studies which have evaluated the relationship of any effects of NIS inhibitors on newborns and/or infants showed no associations between environmental perchlorate exposure and newborn and/or infant thyroid function (21, 22, 25), with one exception. The latter was a study reporting higher newborn SH with high levels of perchlorate in drinking water during pregnancy (20). To the best of our knowledge, the present study is the first to assess the effect of potential co-exposure to all three NIS inhibitors on newborn thyroid function. Thus, our results suggest that co-exposure to maternal NIS inhibitors can negatively affect newborn thyroid health.

We found that median urinary perchlorate concentration in Turkish lactating women (3.89 μg/L) was relatively higher than in the U.S. lactating women [3.0 μg/L (26), 3.1 μg/L (27)]. This result is consistent with our previously published work, in which we evaluated urinary perchlorate concentrations in non-pregnant and non-lactating women and found that median urinary perchlorate concentration (6.4 μg/L) was more than twice as high as the median concentration found in U.S. women (2.9 μg/L) (15, 16). We found similar median concentrations for maternal urinary thiocyanate and nitrate in this study and in our previously published work including non-lactating and non-pregnant women (15). Interestingly, the median maternal urinary thiocyanate concentrations (274 μg/L) in our study were relatively lower than the U.S. lactating women (514 μg/L) (27). We believe this discrepancy is not related to the number of active smokers in the study groups since only a few women actively smoked in both groups during lactation. One possible explanation for this discrepancy is lactating Turkish women smoke with less frequency and intensity compared with U.S. lactating women.

The finding that no significant correlations between maternal TSH and any maternal urinary NIS inhibitors are congruent with studies reporting lack of association between elevated TSH levels and NIS inhibitor exposure (16, 22, 28). However, a cross-sectional study of the US population showed that increased urinary perchlorate was significantly associated with increased TSH levels (29). Possible explanations may account for the discrepancy in the literature are the population demographics and characteristics—e.g., pregnant vs. non-pregnant—genetic differences in NIS metabolism and differences in methodologies.

The median colostrum perchlorate concentration in our study (2.30 μg/L) was remarkably similar to the previously published perchlorate levels found in colostrum collected from lactating U.S. women (30). Our findings were consistent with the median perchlorate levels reported for breast milk by Kirk et al., but lower than the mean perchlorate concentration (10.5 μg/L), indicative of the right-skewed distribution of perchlorate in the Kirk et al. study (28). Different study participants across different studies may have had different perchlorate exposures from differences in perchlorate intake from diet and drinking water. Alternatively, perchlorate secretion into breast milk could change with time: hormonal changes in lactation could affect NIS expression and thus result in differential NIS inhibitor secretion into colostrum vs. breast milk. For example, prolactin mediates iodine accumulation in cultured mammary tissues (31).

The perchlorate intake estimation in newborns revealed that their intake was below the U.S. EPA reference dose of 0.70μg/kg/day (32) and similar to the median perchlorate intake (0.16μg/kg/day) estimated by Valentin-Blasini et al. in U.S. infants (24). We currently found no significant correlation between colostrum perchlorate concentrations and newborn TSH. The reason for this non-significant effect could be that in the first 48 h postpartum may be too early to adequately determine the relationship between colostrum perchlorate concentration and newborn thyroid health status. On the other hand, these levels may be unrelated given Leung et al.'s finding that breast milk perchlorate concentration and infant TSH were unrelated (19).

Although we found no significant relationship between colostrum perchlorate concentrations and newborn TSH, a significant positive correlation was observed between colostrum perchlorate concentrations and maternal TSH levels. Importantly, this finding may provide important evidence that environmental perchlorate exposure is associated with increased production of maternal TSH and, at higher quartiles of co-exposure, leads to increased newborn TSH.

This study provides novel data indicating that lactating study participants have a high intake of some NIS inhibitors and that this may affect newborn thyroid health. However, our study is limited in that maternal urinary iodine concentrations substantially varied between study participants (data not shown), which may be related to usage of iodine-containing disinfectant for skin preparation before delivery. Since we believed different cutaneous absorption of available iodine could have resulted in variations, urinary iodine concentrations were not included in the analysis.

## Conclusion

The present study provides the first indication that co-exposure to three common maternal NIS inhibitors, namely perchlorate, thiocyanate, and nitrate may alter newborn TSH levels. We found that increased newborn TSH levels were associated with higher quartiles of co-exposure to the maternal NIS inhibitors. Our results therefore signify an important first step in the investigation of co-exposure to NIS inhibitors on newborn thyroid function. However, given that colostrum perchlorate concentration appeared to have no effect on newborn thyroid function, further study is warranted. Overall, this study provides critical new insights into the effects of co-exposure to maternal NIS inhibitors on newborn thyroid function. In addition, the results given here have important implications for the evaluation of NIS inhibitor exposure in lactating women and its potential effect on newborn thyroid health status in Turkish populations.

## Author contributions

AO was study PI and involved in all aspects of design, interpretation, and writing. BB led lab analysis and contributed to writing. YU wrote the manuscript with support from AO and BB. MusS, IU, and MuhS helped supervise the project. MuhS and OS contributed to the statistical analysis. LV-B and MM-E involved in lab analysis. PK, MM, and ME were involved in subject selection and coordinated sample collection at the hospital. CA-L, ZK, and CT contributed to sample collection at the hospital. All authors provided critical feedback and helped shape the research, analysis and manuscript.

### Conflict of interest statement

The authors declare that the research was conducted in the absence of any commercial or financial relationships that could be construed as a potential conflict of interest.
